# Supplementary figures and images for: Genome-Enhanced Detection and Identification (GEDI) of plant pathogens
Source: PeerJ. 2018 Feb 22;6:e4392. doi: 10.7717/peerj.4392 (PMC5825881; doi:10.7717/peerj.4392)

Supplementary Figures

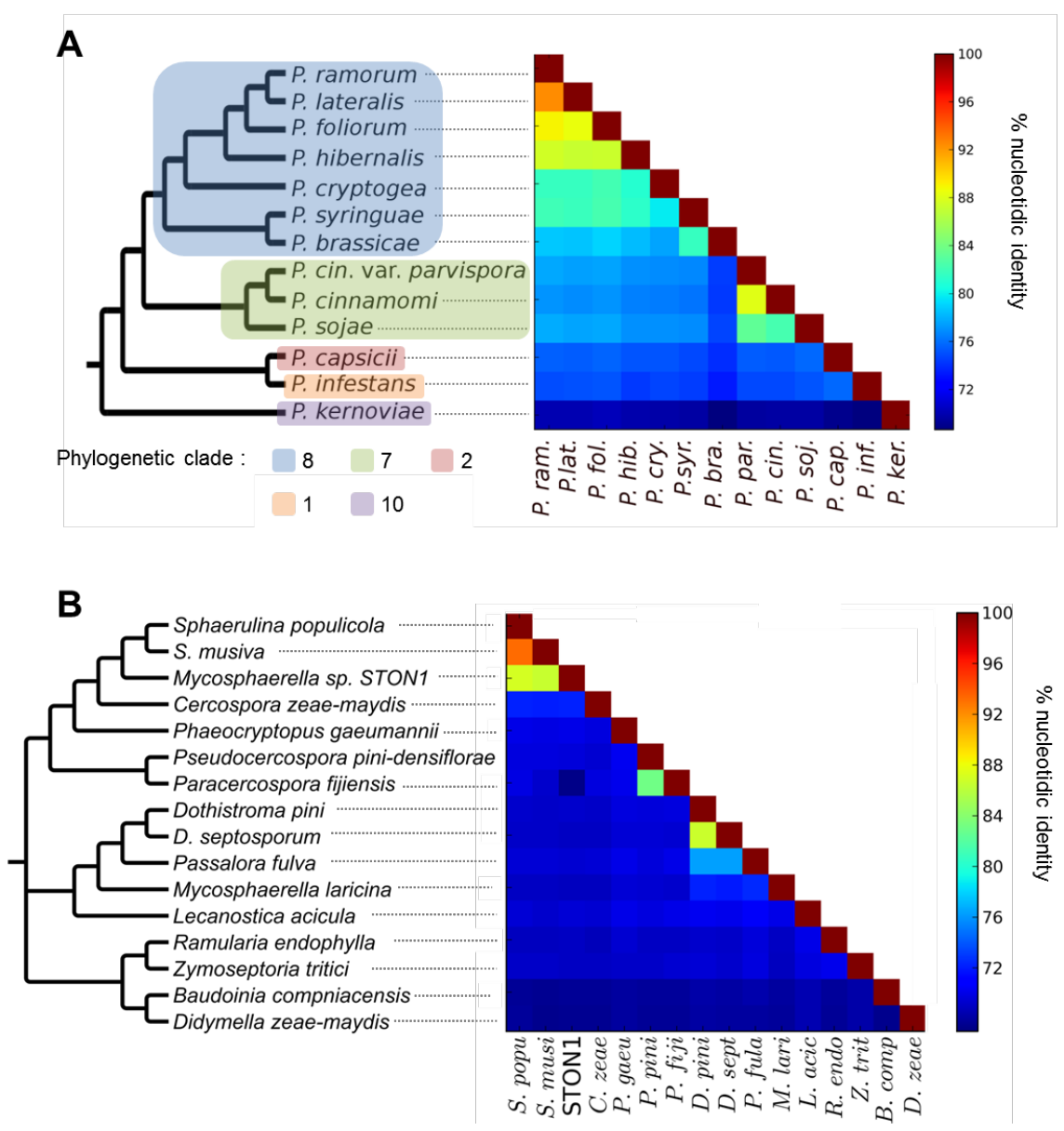

Supplement: Figure S1 — Neighbor-joining trees on the left were reconstructed from the matrix of nucleotidic identity between pairs of genomes presented on the right. For the Phytophthora dataset, the phylogenetic clades as defined in Blair et al., 2008 are indicated on the phylogenetic tree. [file peerj-06-4392-s001.pdf]

**A**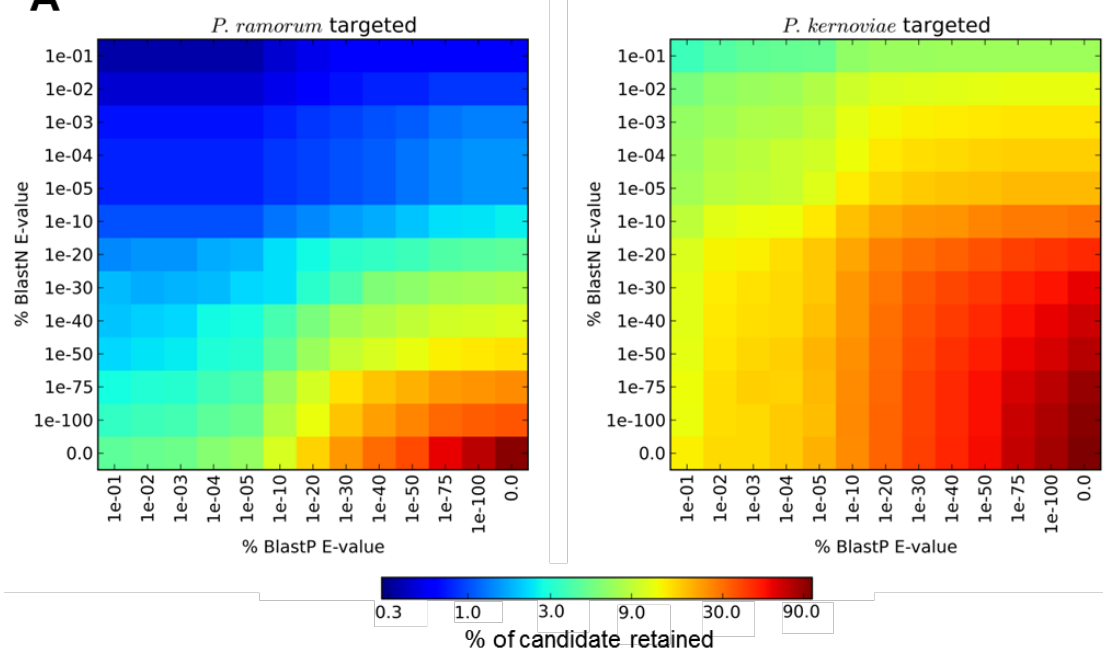**B**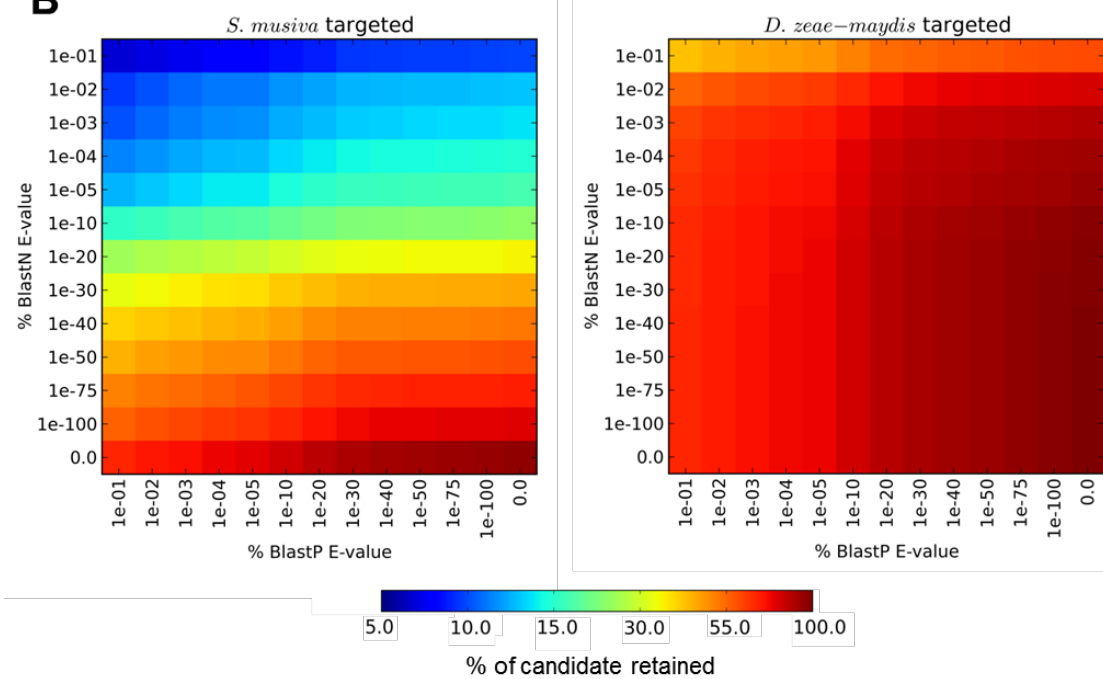

Supplement: Figure S3 — (A) Filtering results for Phytophthora ramorum and P. kernoviae used as target species with the Phytophthora dataset; (B) filtering results for Sphaerulina musiva and Didymella zeae-maydis used as target species with the Dothideomycetes dataset. [file peerj-06-4392-s003.pdf]
