# Supplementary material for: Genome-Enhanced Detection and Identification (GEDI) of plant pathogens
Source: PeerJ. 2018 Feb 22;6:e4392. doi: 10.7717/peerj.4392 (PMC5825881; doi:10.7717/peerj.4392)
Supplement: Figure S2 — (A) Unique candidates as predicted in Module 2 of the pipeline (OrthoMCL search); (B) unique candidates retained after Module 3 (filtering with BLASTp and BLASTn with e-value cutoff of 1e–05). [file peerj-06-4392-s002.pdf]

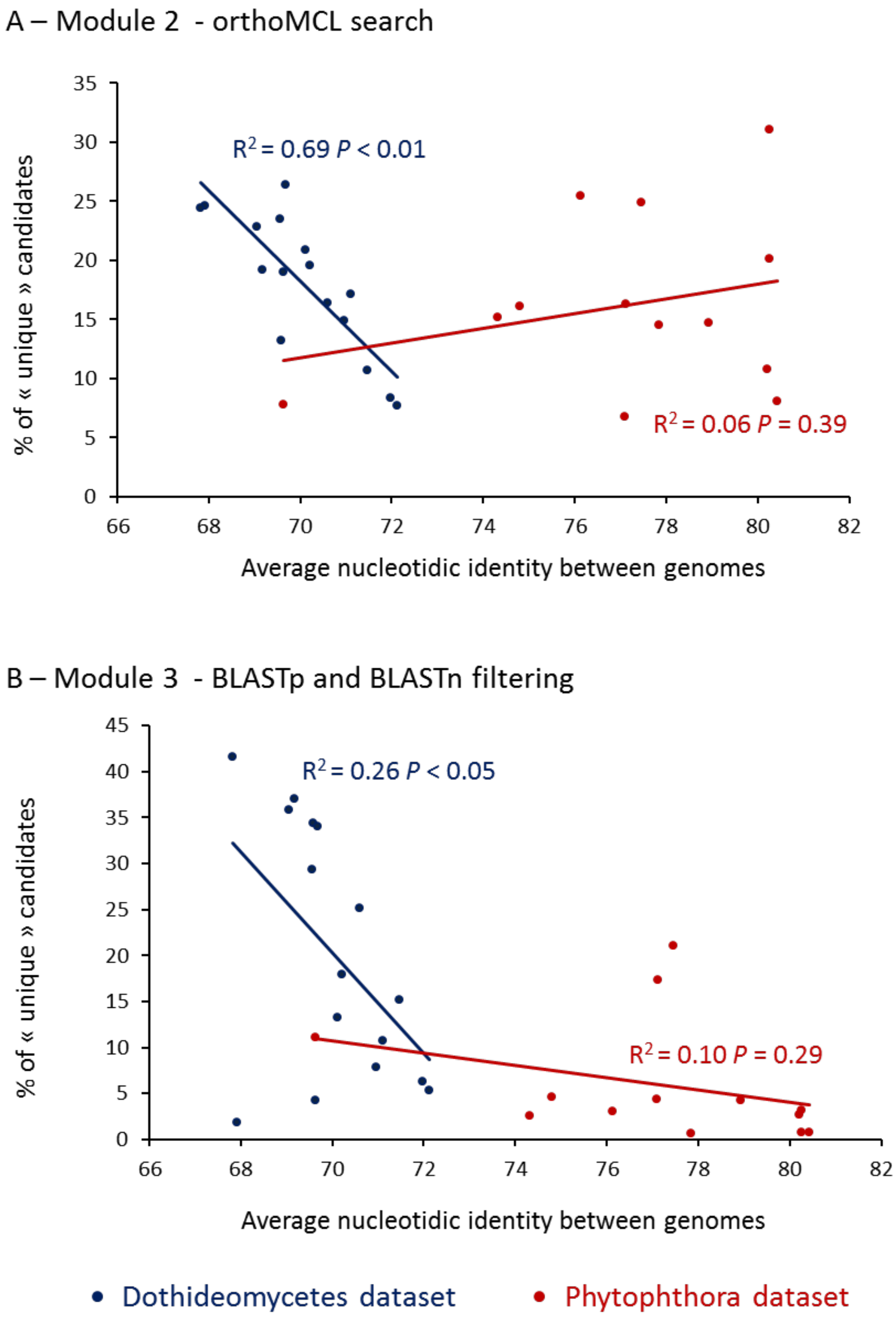

Figure S2. **Relationships between the proportion of "unique" candidates and the average nucleotidic identity between genomes in the Phytophthora and Dothideomycetes datasets.** A) Unique candidates as predicted in Module 2 of the pipeline (OrthoMCL search); B) unique candidates retained after Module 3 (filtering with BLASTp and BLASTn with e-value cutoff of 1e-05).
