# Supplementary material for: Genome-Enhanced Detection and Identification (GEDI) of plant pathogens
Source: PeerJ. 2018 Feb 22;6:e4392. doi: 10.7717/peerj.4392 (PMC5825881; doi:10.7717/peerj.4392)
Supplement: Supplemental Information 4 — Table S1. Assembly statistics and gene content for the Dothideomycete genome sequences generated or downloaded in this study. Table S2. Assembly statistics and gene content for the rust genome sequences generated or downloaded in this study. Table S3. Assembly statistics and gene content for the Phytophthora genome sequences generated or downloaded in this study. [file peerj-06-4392-s004.docx]

Table S1. Assembly statistics and gene content for the Dothideomycete genome sequences generated or downloaded in this study

|  |  |  | Source | Genome assembly accession | Genome coverage | | Total size (Mpb) | | | # of scaffolds | N50 (Mb) | BUSCO coverage | # of predicted gene models |
| --- | --- | --- | --- | --- | --- | --- | --- | --- | --- | --- | --- | --- | --- |
| Capnodiales | | |  |  |  | | |  | |  |  |  |  |
|  | *Dothistroma pini* | | TAIGA | GCA_002116355.1 | 1264.0x | | 30.0 | | | 642 | 0.13 | 1436 (99.9%) | 12,610 |
|  | *Lecanostica acicula* [=*M. dearnessii*] | | TAIGA | GCA_000504345.1 | 295.0x | | 34.8 | | | 2052 | 0.18 | 1436 (99.9%) | 13,049 |
|  | *Pseudocercospora pini-densiflorae* [=*M. gibsonii*] | | TAIGA | GCA_000504365.1 | 304.0x | | 45.2 | | | 5537 | 0.08 | 1434 (99.7%) | 16,709 |
|  | *Mycosphaerella laricina* | | TAIGA | GCA_000504385.1 | 527.0x | | 26.6 | | | 642 | 0.14 | 1435 (99.8%) | 10,387 |
|  | *Ramularia endophylla* [=*M. punctiformis*] | | TAIGA | GCA_002116395.1 | 926.0x | | 41.3 | | | 3592 | 0.06 | 1432 (99.6%) | 13,120 |
|  | *Mycosphaerella* sp. STON1 | | TAIGA | GCA_000504405.1 | 491.0x | | 27.5 | | | 1929 | 0.07 | 1428 (99.3%) | 9709 |
|  | *Passalora fulva* [=*Cladosporium fulvum*] | | DOE-JGI | GCA_000301015.1 | 21.0x | | 61.1 | | | 4864 | 0.06 | 1417 (98.5%) | 14,127 |
|  | *D. septosporum* [=*M. pini*] | | DOE-JGI | GCA_000340195.1 | 34.28x | 30.2 | | | 20 | | 2.6 | 1435 (99.8%) | 12,580 |
|  | *Zymoseptoria tritici* [=*M. graminicola*] | | DOE-JGI | GCA_000219625.1 | 8.9x | | 39.7 | | | 21 | 2.7 | 1434 (99.7%) | 10,952 |
|  | *Paracercospora fijiensis* [=*M. fijiensis*] | | DOE-JGI | GCA_000340215.1 | 7.11x | | 74.1 | | | 56 | 5.9 | 1433 (99.7%) | 13,107 |
|  | *Sphaerulina populicola* [= *M. populicola*] | | DOE-JGI | GCA_000291705.1 | 18x | | 33.2 | | | 502 | 0.25 | 1430 (99.4%) | 9739 |
|  | *Sphaerulina musiva* [=*M. populorum*] | | DOE-JGI | GCA_000320565.2 | 35x | | 29.3 | | | 72 | 2.04 | 1429 (99.4%) | 10,233 |
|  | *Baudoinia compniacensis* | | DOE-JGI | NA | 43.5x | | 21.8 | | | 19 | 1.3 | 1434 (99.7%) | 10,513 |
|  | *Cercospora zeae-maydis* | | DOE-JGI | NA | 39.3x | | 46.6 | | | 917 | 0.72 | 1436 (99.9%) | 12,020 |
| Dothideales | | |  |  |  | | | | | |  |  |  |
|  | *Phaeocryptopus gaeumannii* [*=Adelopus gaeumannii*] | | TAIGA | GCA_002116385.1 | 1016.0x | | 34.0 | | | 737 | 0.14 | 1433 (99.7%) | 14,295 |
| Pleosporales | | |  |  |  | |  | | |  |  |  |  |
|  | *Didymella zeae-maydis* | | DOE-JGI | NA | 1x | | 32.6 | | | 2072 | 0.07 | 1438 (100%) | 10,299 |

Table S2. Assembly statistics and gene content for the rust genome sequences generated or downloaded in this study

|  |  | |  | Source | | Genome assembly accession | Genome coverage | Total size (Mpb) | # of scaffolds | N50 | BUSCO coverage | # of predicted gene models |
| --- | --- | --- | --- | --- | --- | --- | --- | --- | --- | --- | --- | --- |
| Uredinales | | | | |  |  |  | | | | | |
|  | Melampsoraceae | | | |  |  |  | | | | | |
|  |  | *Melampsora alli-populina* | | TAIGA | |  |  |  |  |  |  | 11,682 |
|  |  | *M. larici-populina* | | DOE-JGI | | GCA_000204055.1 | 8.4x | 101.1 | 462 | 1.1 | 682 (47.4%) | 16,399 |
|  |  | *M. medusae* f. sp *deltoides* | | TAIGA | |  |  | 92.3 |  | 0.006 | 569 (39.6%) | 25,813 |
|  |  | *M. occidentalis* | | TAIGA | |  |  | 109.8 |  | 0.004 | 628 (43.7%) | 21,406 |
|  |  | *M. abietis-canadensis* | | TAIGA | |  |  | 83.6 |  | 0.022 | 662 (46.0%) | 17,309 |
|  |  | *M. aecidioides* | | TAIGA | |  |  | 87.4 |  | 0.001 | 331 (23.0%) | 22,170 |
|  |  | *M. pinitorqua* | | TAIGA | | GCA_000464645.1 | 959.0x | 34.0 | 12,325 | 0.005 | 287 (20.0%) | 4879 |
|  | Cronartiaceae | | | |  |  |  | | | | | |
|  |  | *Cronartium ribicola* | | TAIGA | | GCA_000500245.1 | 629.0x | 94.3 | 41,096 | 0.004 | 941 (65.4%) | 12,010 |
|  |  | *C. quercum* f. sp. *fusiforme* | | DOE-JGI | | NA | 70.4x | 76.6 | 1,198 | 0.31 | 929 (64.6%) | 13,903 |
|  |  | *Endocronartium harknesii* | | TAIGA | | GCA_000500795.1 | 671.0x | 56.9 | 25,178 | 0.003 |  | 4180 |
|  |  | *C. comandrae* | | TAIGA | | GCA_000464975.1 | 549x | 68.6 | 35,717 | 0.002 | 289 (20.1%) | 3026 |
| Pucciniales | | | | |  |  |  | | | | | |
|  | Pucciniaceae | | | |  |  |  | | | | | |
|  |  | *Puccinia graminis* f. sp. *tritici* | | Broad | | GCA_000149925.1 | NA | 88.7 | 393 | 0.96 | 1060 (73.7%) | 20,534 |
|  |  | *P. triticina* | | Broad | | GCA_000151525.2 | 31.0x | 135.3 | 14,818 | 0.54 | 1141 (79.3%) | 15,685 |
|  |  | *P. striiformis* | | Broad | | GCA_001191645.1 | 81.0x | 117.4 | 9716 | 0.52 | 744 (51.7%) | 20,482 |
| Mixiales | | | | |  |  |  | | | | | |
|  | Mixiaceae | | | |  |  |  | | | | | |
|  |  | *Mixia osmundae* | | DOE-JGI | | GCA_000708205.1 | 150.9x | 13.6 | 156 | 1.2 | 1340 (93.2%) | 6903 |
| Sporidiobolales | | | | |  |  |  | | | | | |
|  | Incertae sedis | | | |  |  |  | | | | | |
|  |  | *Sporobolomyces roseus* | | DOE-JGI | | NA |  |  |  |  | 1280 (89.0%) | 5536 |
| Sporidiales | | | | |  |  |  | | | | | |
|  | Incertae sedis | | | |  |  |  | | | | | |
|  |  | *Rhodotorula graminis* | | DOE-JGI | | GCA_001329695.1 | 8.55x | 21.0 | 26 | 1.42 | 1336 (92.9%) | 7283 |

Table S3. Assembly statistics and gene content for the *Phytophthora* genome sequences generated or downloaded in this study

|  |  |  | Source | | Genome assembly accession | Genome coverage | Total size (Mpb) | | # of scaffolds | N50 | | BUSCO coverage | # of predicted gene models | |
| --- | --- | --- | --- | --- | --- | --- | --- | --- | --- | --- | --- | --- | --- | --- |
| Clade 1 | | | |  |  |  | |  |  | |  |  |  |  |
|  | *P. infestans* | | Broad Institute | | GCA_000142945.1 | NA | 228.5 | | 4921 | 1.5 | | 348 (81.1%) | 18,140 | |
| Clade 2 | | |  | |  |  |  | |  |  | |  |  | |
|  | *P. capsici* | | DOE-JGI | | GCA_000325885.1 | 35.0x | 56.0 | | 10,750 | 0.70 | | 351 (81.8%) | 19,805 | |
| Clade 7 | | | |  |  |  | |  |  | |  |  |  |  |
|  | *P. sojae* | | DOE-JGI | | GCA_000149755.2 | 8x | 82.6 | | 82 | 7.6 | | 353 (82.3%) | 26,584 | |
|  | *P. cinnamomi* var*. cinnamomi* | | DOE-JGI | | NA | 69.6x | 78.0 | | 1314 | 0.26 | | 349 (81.4%) | 26,131 | |
| Clade 8 | | | |  |  |  | |  |  | |  |  |  |  |
|  | *P. ramorum* | | | DOE-JGI | GCA_000149735.1 | NA | | 66.7 | 2576 | | 0.3 | 350 (81.6%) | 15,743 |  |
|  | *P. lateralis* | | TAIGA | | GCA_000500205.2 | 470.0x | 52.4 | | 9039 | 0.023 | | 353 (82.3%) | 17,533 | |
|  | *P. hibernalis* | | | Tyler’s lab | NA | NA | | 71.2 | 6587 | | 0.022 | 344 (80.2%) | 20,209 |  |
|  | *P. foliorum* | | Tyler’s lab | | NA | NA | 49.0 | | 5320 | 0.016 | | 339 (79.0%) | 15,849 | |
|  | *P. syringae* | | Tyler’s lab | | NA | NA | 57.0 | | 6572 | 0.016 | | 352 (82.1%) | 18,201 | |
|  | *P. brassicae* | | Tyler’s lab | | NA | NA | 72.8 | | 12,447 | 0.012 | | 350 (81.6%) | 22,241 | |
|  | *P. cryptogea* | | TAIGA | | GCA_000468175.1 | 345.0x | 103.0 | | 20,849 | 0.012 | | 347 (80.9%) | 30,812 | |
| Clade 10 | | |  | |  |  |  | |  |  | |  |  | |
|  | *P. kernoviae* | | TAIGA | | GCA_000448265.2 | 474.0X | 39.4 | | 5,026 | 0.064 | | 344 (80.2%) | 10,012 | |
