# Supplementary material for: Genome-Enhanced Detection and Identification (GEDI) of plant pathogens
Source: PeerJ. 2018 Feb 22;6:e4392. doi: 10.7717/peerj.4392 (PMC5825881; doi:10.7717/peerj.4392)
Supplement: Text S1 [file peerj-06-4392-s005.docx]

Supplementary material

MATERIAL AND METHODS

RNAseq libraries

To help in *Ab initio* gene and protein model predictions for Dothideomycetes and Phytophthora genomes obtained in the TAIGA project (Table S1 and S3), RNA-seq libraries from were constructed from mycelium obtained from the same isolates than the ones used in the genome sequencing.

**Dothideomycete genomes.** Each culture was grown for 48h in liquid Malt Extract (2%) media, Yeast Nitrogen Base (YNB) + 2% Glucose and YNB + 0.15% Asparagine, before harvesting the mycelium by centrifugation. Total RNA was purified with the RNeasy Mini Kit following N2 grinding. Total RNA was used to purify polyA^+^RNA fraction using the MACS mRNA Isolation Kit (Miltenyi Biotec, Germany) following the manufacturer’s instructions. Double-stranded cDNA was synthesized from the purified polyA^+^RNA using Superscript Double-Stranded cDNA Synthesis kit (Invitrogen, USA) and random hexamer primers (Invitrogen) at a concentration of 5µM. The cDNA sheared using Covaris E110 (Covaris, USA) for 75 second at “Duty cycle” of 20% and “Intensity” of 5, and 200-300bp fraction was excised from 8% PAGE, and a PET library was prepared by following the pair end library preparation protocol. Briefly, the DNA was subject to end-repair, and phosphorylation by T4 DNA polymerase, Klenow DNA Polymerase, and T4 polynucleotide kinase respectively in a single reaction, and then 3’ A overhangs generation by Klenow fragment (3’ to 5’ exo minus), and ligated to Illumina PE adapters, which contain 5’ T overhangs. The adapter-ligated products were purified, then PCR-amplified with Phusion DNA Polymerase in 10 to 15 cycles using Illumina’s primer set (Illumina). PCR product of desired size range was purified using a 8% PAGE, and the DNA quality was assessed and quantified using an Agilent DNA 1000 series II assay and Quant-iT dsDNA HS Assay Kit using Qubit fluorometer (Invitrogen), then diluted to 8nM. The final concentration was double checked and determined by Quant-iT dsDNA HS Assay again for Illumina Sequencing.  Libraries were pooled and run on 75 base indexed PET HiSeq 2000 lanes at Canada's Michael Smith Genome Sciences Centre or GSC (Vancouver, Canada).

**Phytophthora genomes***.* Phytophthora cultures were grown for 48h in Potatoes dextrose broth and liquid V8 media, before harvesting the mycelium by centrifugation. Total RNA was quantified using a NanoDrop Spectrophotometer ND-1000 (NanoDrop Technologies, Inc.) and its integrity was assessed using a 2100 Bioanalyzer (Agilent Technologies). Libraries were generated from 250 ng of total RNA using the TruSeq stranded mRNA Sample Preparation Kit (Illumina), as per the manufacturer’s recommendations. Libraries were quantified using the Quant-iT™ PicoGreen® dsDNA Assay Kit (Life Technologies) and the Kapa Illumina GA with Revised Primers-SYBR Fast Universal kit (D-Mark). Average size fragment was determined using a LaChip GX (PerkinElmer) instrument. Libraries were pooled and sequenced on 75 base indexed PET HiSeq 2000 lanes at McGill University and Génome Québec Innovation Centre.

Nucleotidic identity between genomes

To measure average nucleotidic identity between pairwise species within groups, scaffold from each genome pairs were aligned by using the progressiveMauve algorithm of the Mauve genome aligner (ver. 2.4.0) (1). Alignment outputs (fasta) were then parsed with a custom Python program (available upon request) to estimate pairwise nucleotidic identity. T-REX (2) was then used to reconstruct NJ tree based on the matrix of nucleotidic identity values.

Filtering simulations

False positive filtering with BLASTn and BLASTp (Module 3 of the pipeline) was tested with a first set of 11 *Phytophthora* genomes and a second set of 16 Dothideomycetes genomes. Module 2 (orthoMCL) was first run independently on both sets with default parameters (Blastp e-value of 1e-20, minimum sequence overlap of 50% and MCL inflation parameter of 1.5). In a first simulation round, we filtered the “unique” orthoMCL candidates obtained for each taxa of the two dataset with the e-value cutoff of the BLASTP and BLASTn algorithms set at 1e-05. As a second simulation, Module 3 was run on the two sets by targeting two taxa for each set: the taxa showing the lowest average identity with other taxa and the taxa with the highest average distance. Thus, for the Phytophthoras, *P. ramorum* (average identity of 80.4% [±5.1]) and *P. kernoviae* (69.6% [± 0.4]) were selected as targets (Fig. S1A); for the Dothideomycetes, the target taxa were *Sphaerulina musiva* (71.98% [±4.8]) and *D. zeae* (67.83% [±0.2]) (Fig. S2A). For each target taxa, the number of candidates obtained in Module 2 were filtered using all pairwise combinations of BLASTn and BLASTp e-value cutoffs from a list of 13 e-value cutoff ranging from 1e-01 to 0.0 (169 combinations).

RESULTS

We ran Module 2 of the pipeline (orthoMCL) on a first genomes dataset of 11 taxa and a second one with 16 Dothideomycetes taxa. Genomic identity between taxon pairs was in the same range for the two datasets (67 to 93%); however pairwise distance values were distributed all over this range for the Phytophthora, whereas for the Dothideomycetes a large majority of the distances (90%) were in lowest quartile (values ranging between 67 and 72%) of the distribution (Fig. S1A and B). For the Dothideomycete dataset we found a significant negative correlation between the number of “unique” orthoMCL candidates and the level of genomic identity between taxa (Fig. S2A), indicating that the use of closely related species as target and non-target taxa will reduce the number of “unique” candidates. In contrast, the same relationship was positive and non-significant with the Phytophthora dataset (Fig. S2A). This difference between the two datasets could be related to the quality of the *de novo* genome assemblies that were considered for the Phytophthora dataset. Indeed, scaffold N50-values for the Phytophthora genomes were generally lower (average = 0.27Mb ±1.22; median value =0.016Mb) than those obtained for the Dothideomycetes genomes (ave. = 1.03Mb ±1.18; median value =0.14Mb), suggesting a higher fragmentation level for some of the *Phytophthora* genomes (Table S1 and S3). Genome fragmentation may cause premature truncation of protein-coding sequence by the termination of a scaffold in the middle of an open reading frame; presence of these fragments will likely causes some errors in the inference of taxa-specific genes with clustering algorithms such as orthoMCL.

We then looked at the number of “unique” candidates retained for each taxa with both BLASTp and BLASTn filters set to the cutoff value of 1e-05. The negative relationship between genomic identity and number of “unique” candidate previously observed for the Dothideomycetes was retrieved (Fig. S2B). Interestingly, we also found a slight negative correlation between these two factors for the Phytophthoras (Fig. S2B), suggesting that BLASTp and BLASTn filtering likely helped in reestablishing the relation between the number of “unique” candidate found in each taxon and the phylogenetic distance between these taxa. To further explore this possibility we selected in each dataset the taxa showing the lowest average identity with all other taxa of the dataset and the taxa with the highest average distance and used them as target species. We then tested 169 combinations of BLASTp and BLASTn e-value cutoffs to filter out false positive “unique” orthoMCL candidates obtained for these targeted taxa. Overall, when the genomic identity between target and non-target species is low, e-values used for the BLASTp and BLASTn cutoffs don’t seem to influence drastically the number of “unique” candidates retained (i.e. searches for *P. kernoviae* and *D. zeae-maydis*) (Fig. S3A and B). In contrast, this variation was more pronounced when target and non-target species were less distant (i.e. cases with *P. ramorum* and *S. musiva*), underlying the importance of applying stringent filters when closely related species are considered for search of “unique” gene candidates. More specifically, tests carried out on the Phytophthora dataset indicated that both the BLASTn and BLASTp filters acted the same in rejecting potential false positive candidates, either when the species targeted and non-targeted were closely related (i.e. *P. ramorum*) or not (*P. kernoviae*) (Fig. S3A). Conversely, simulations with the Dothideomycetes indicated a substantial effect of the BLASTn filter on the number of candidates rejected, compared to a limited effect obtained with the BLASTp filter (Fig. S3B). This trend was particularly pronounced when the genomic distances between the target and the non-target species were short (i.e. *S. musiva*). The BLASTp filter querying “unique” candidates against non-target protein sets aims to minimize errors due to the presence of truncated protein found in fragmented *de novo* assemblies, likely explaining its efficiency in filtering out candidates predicted from genomes of lower quality as those included into the Phytophthora dataset.

REFERENCES

1. **Darling ACE**, **Mau B**, **Blattner FR**, **Perna NT**. 2004. Mauve: multiple alignment of conserved genomic sequence with rearrangements. Genome Res **14**:1394–403.

2. **Boc A**, **Diallo AB**, **Makarenkov V**. 2012. T-REX: a web server for inferring, validating and visualizing phylogenetic trees and networks. Nucleic Acids Res **40**:W573–W579.

3. **Blair JE**, **Coffey MD**, **Park SY**, **Geiser DM**, **Kang S**. 2008. A multi-locus phylogeny for *Phytophthora* utilizing markers derived from complete genome sequences. Fungal Genet Biol **45**:266–277.

Supplementary Figures


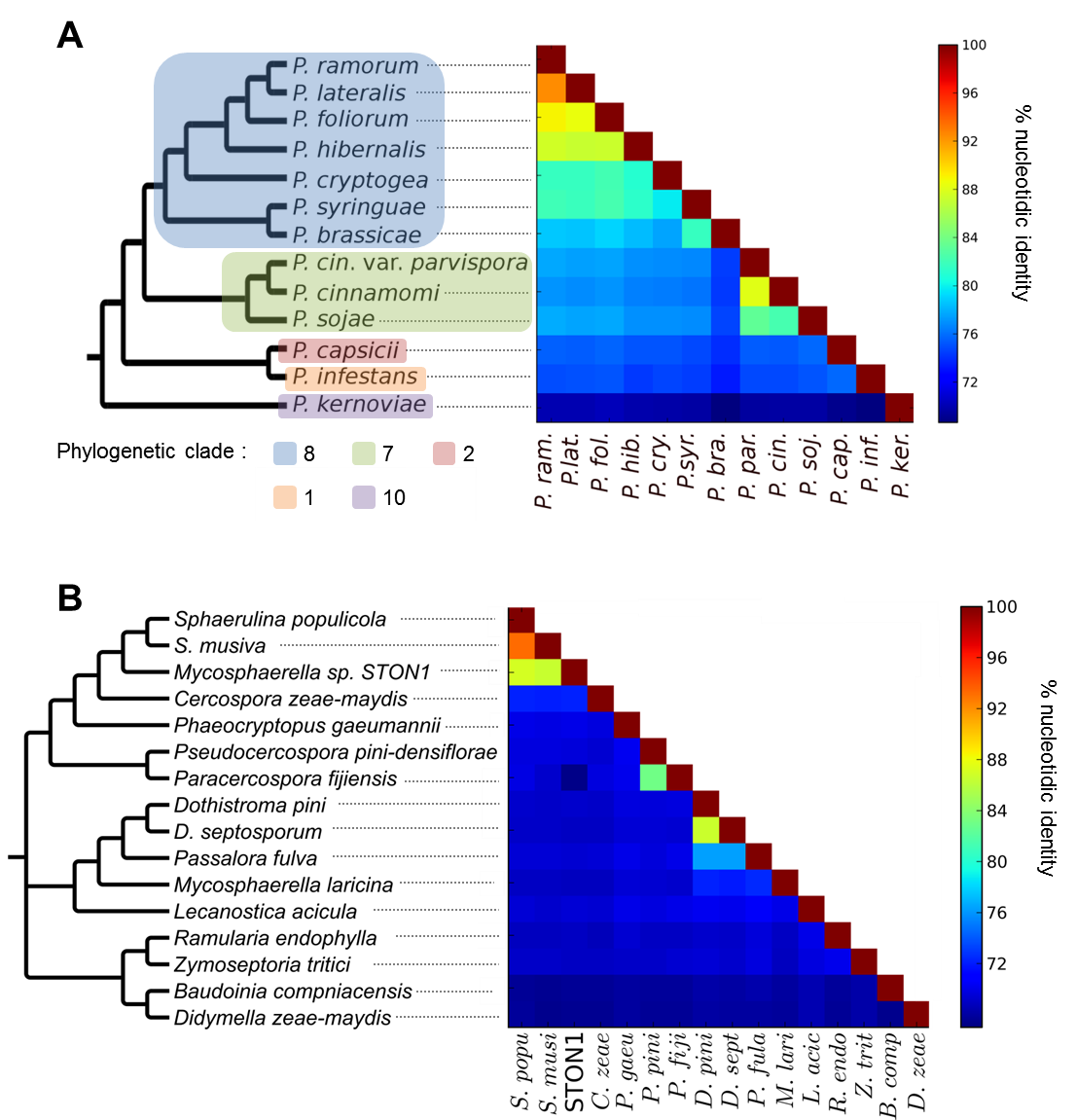


Figure S1. **Nucleotidic identity between pairwise genomes with (A) 11 taxa of Phytophthora and (B) 16 taxa of Dothideomycete**. Neighbor Joining-trees on the left were reconstructed from the matrix of nucleotidic identity between pairs of genomes presented on the right. For the Phytophthora dataset, the phylogenetic clades as defined in (3) are indicated on the phylogenetic tree.


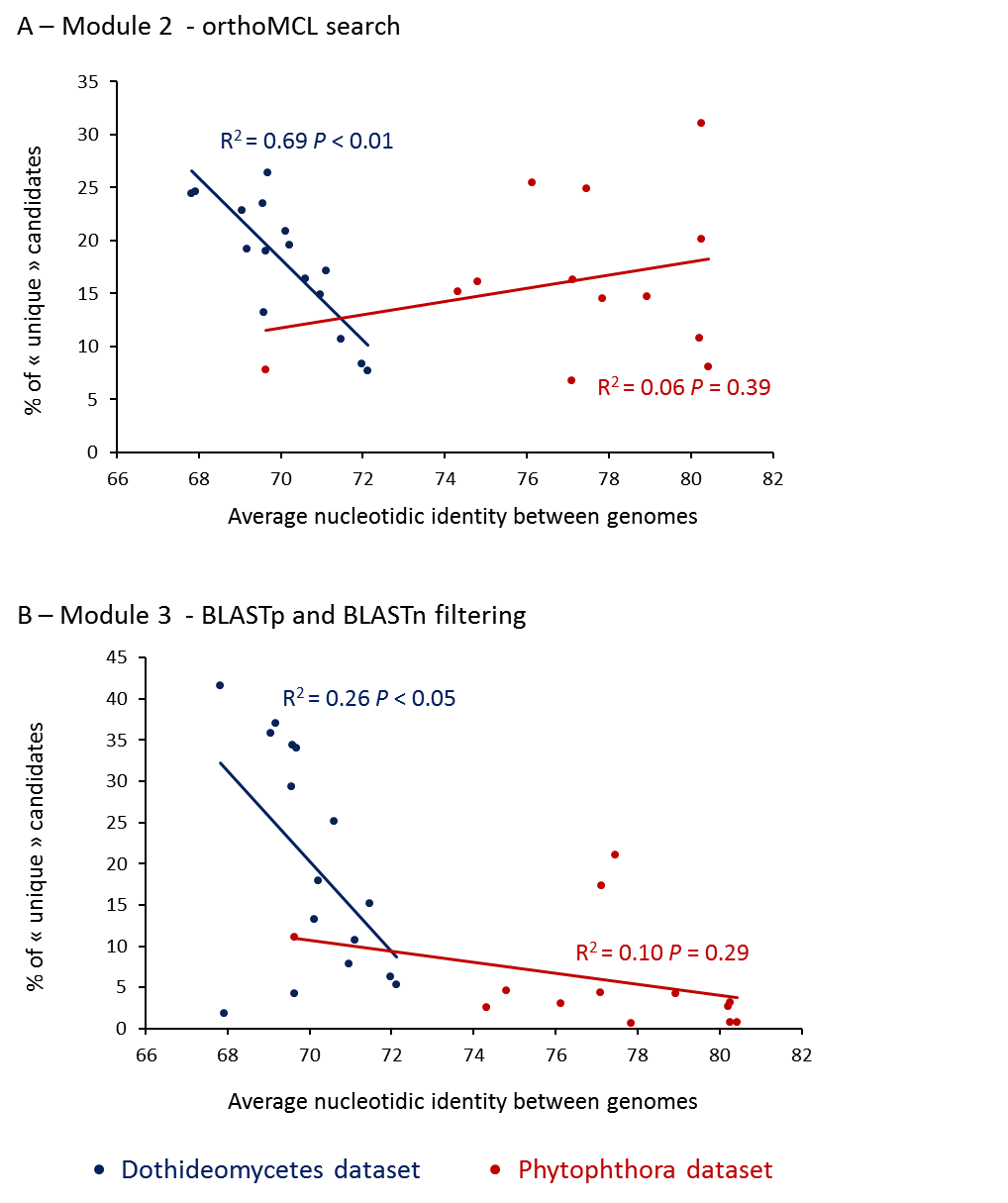


Figure S2. **Relationships between the proportion of “unique” candidates and the average nucleotidic identity between genomes in the Phytophthora and Dothideomycetes datasets.** A, unique candidates as predicted in Module 2 of the pipeline (OrthoMCL search); B, unique candidates after Module 3 (filtering with BLASTp and BLASTn with e-value cutoff of 1e-05).


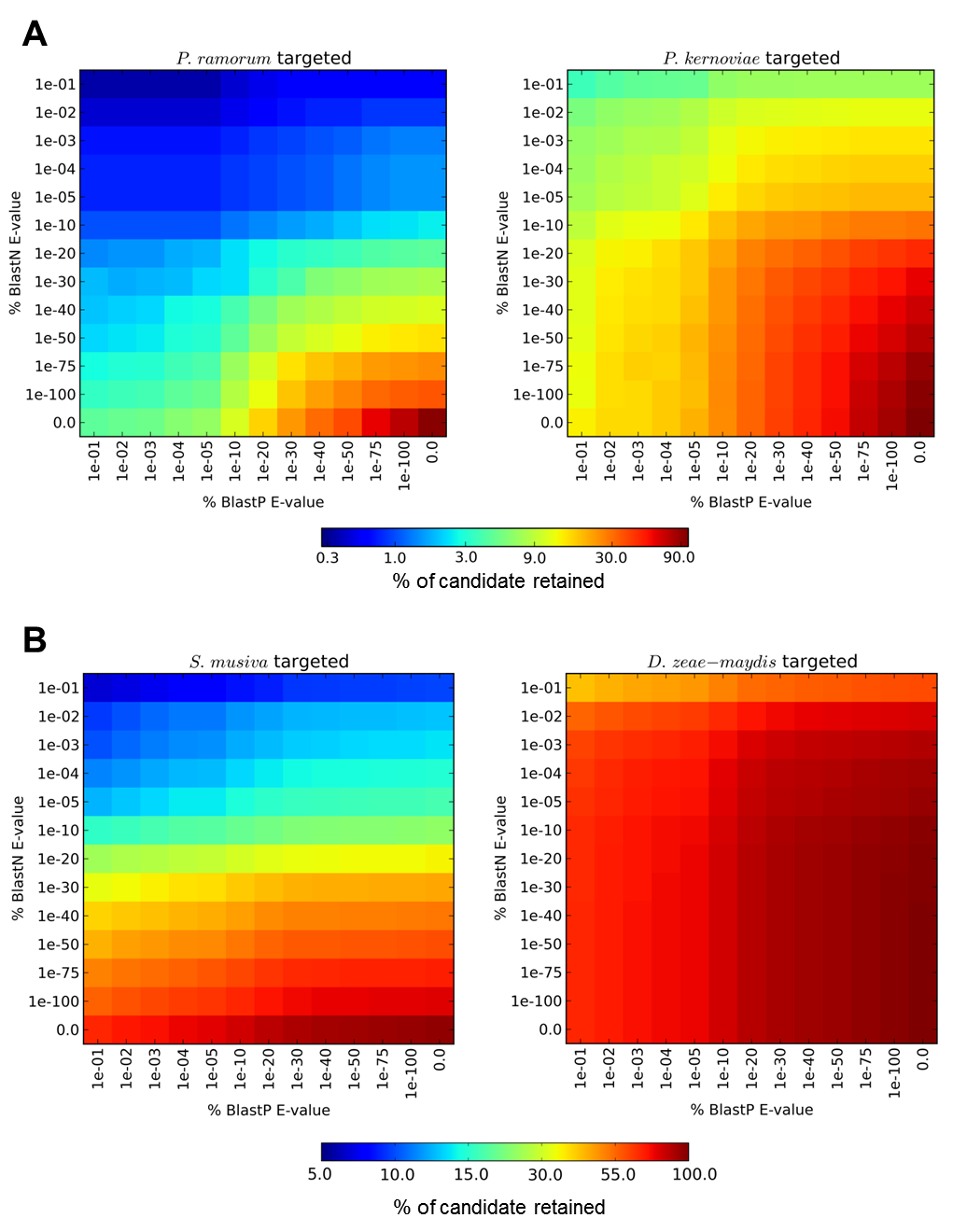


Figure S3. **Proportions of “unique” candidate retained after BLASTp and BLASTn filtering according to different e-value cutoffs.** A) filtering results for *Phytophthora ramorum* and *P. kernoviae* used as target species with the Phytophthora dataset; B) filtering results for *Sphaerulina musiva* and *Didymella zeae-maydis* used as target species with the DOthideomycetes dataset.


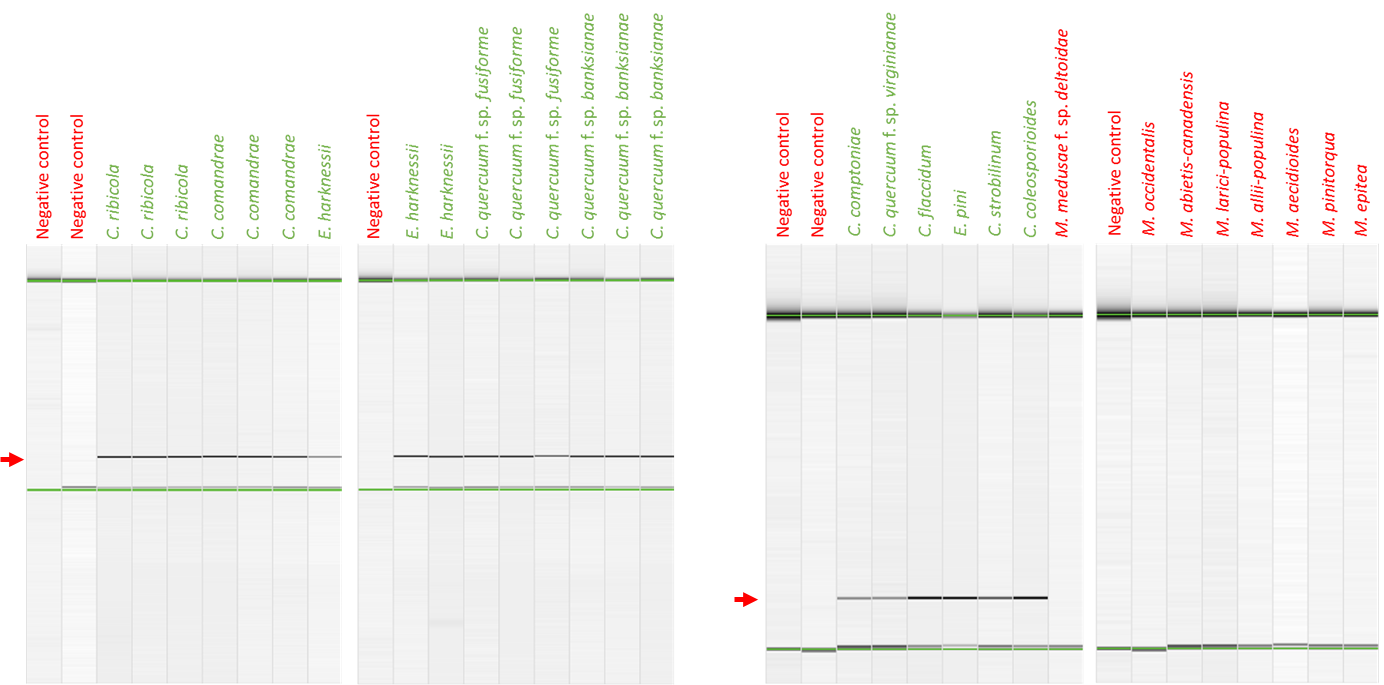


Figure S4. Specificity of the genus-assay *Cronartium*-46. PCR products migrated through a physical gel within capillaries, using the QIAxcel system ; The data output is available as a virtual agarose gel electrophoresis-like image. Target samples are indicated in green and non-target ones in red. Red arrow indicates the position of the expected PCR product.


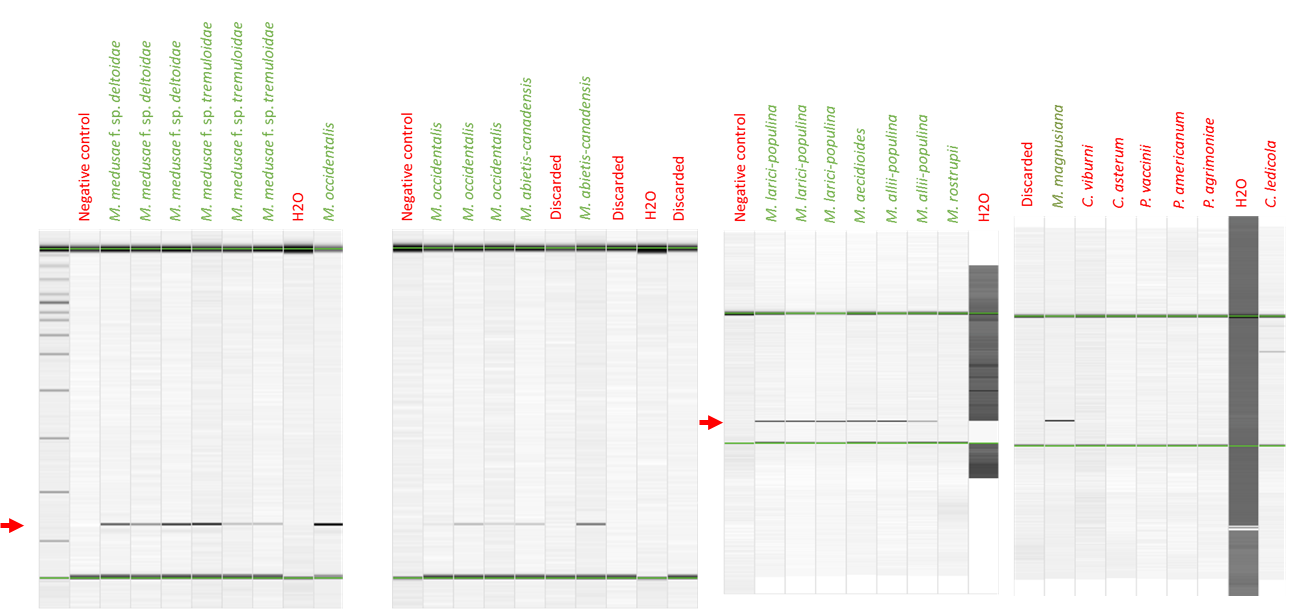


Figure S5. Specificity of the genus-assay *Melampsora*-100. PCR products migrated through a physical gel within capillaries, using the QIAxcel system ; The data output is available as a virtual agarose gel electrophoresis-like image. Target samples are indicated in green and non-target ones in red. Red arrow indicates the position of the expected PCR product.
